# The Acute Effect of Methylphenidate Ingestion in a Visuomotor Task at Absolute Power Alpha in Healthy Subjects

**DOI:** 10.1055/s-0046-1817036

**Published:** 2026-03-23

**Authors:** Vinicius Lourenço, Carlos Amoroso, Renan Vicente, André Azevedo, Eduardo Nicoliche, Bruna Velasques, Marcelo Nobre, Mauricio Cagy, Pedro Ribeiro, Henning Budde, Alex Vasconcelos, Renato Fonseca, Giovanna Zanchetta, Marco Orsini, Marcos Machado, Sávio Barreto, Silmar Teixeira, Victor Hugo Bastos, Francisco Victor Marinho, Isabelle Fernandes, Egídio Nardi

**Affiliations:** 1 Universidade Federal do Rio de Janeiro, Instituto de Psiquiatria, Rio de Janeiro RJ, Brazil.; 2 Universidade Federal do Rio de Janeiro, Escola de Educação Física e Desportos, Rio de Janeiro RJ, Brazil.; 3 Escola Superior de Guerra, Rio de Janeiro RJ, Brazil.; 4 Universidade Federal do Rio de Janeiro, Coordenação dos Programas de Pós-Graduação e Pesquisa de Engenharia, Programa de Engenharia Biomédica, Rio de Janeiro RJ, Brazil.; 5 Medical School Hamburg, Faculty of Human Sciences, Hamburg, Germany.; 6 Reykjavik University, Department of Sport Science, Reykjavik, Iceland.; 7 Universidade Federal Fluminense, Hospital Universitário Antônio Pedro, Niterói RJ, Brazil.

**Keywords:** Methylphenidate, Alpha Rhythm, Electroencephalography, Attention, Psychomotor Performance

## Abstract

**Background:**

Methylphenidate (MPH) is a psychostimulant widely used to enhance attention and executive functions through increased dopaminergic and noradrenergic transmission. Although its effects on cognitive performance are well documented, its acute influence on cortical oscillatory activity, particularly α power, during tasks requiring simultaneous motor and cognitive processing remains poorly understood in healthy adults.

**Objective:**

To investigate the acute effects of 10 mg MPH on absolute α power (8–12 Hz) in frontal regions during a visuomotor task in healthy subjects.

**Methods:**

A total of 13 right-handed healthy adults (7 men; age 25.6 ± 4.5 years) participated in a randomized, double-blind, placebo-controlled, crossover study. A 20-channel electroencephalography (EEG) was recorded before and after execution of the MIRA visuomotor task (joystick response when a moving target crosses a previously memorized position) under placebo and MPH (10 mg) conditions, with sessions 1 week apart. Absolute α power was compared using two-way ANOVA (condition vs. moment: pre- vs. post-joystick press), followed by paired *t* -tests when appropriate.

**Results:**

Significant condition versus moment interactions were observed at F3 ( *p* = 0.006), F4 ( *p* = 0.003), and F8 ( *p* = 0.023). The main effects of condition and/or moment occurred at Fp1, Fp2, and Fz (all *p* < 0.05). The use of MPH attenuated or reversed the typical task-related α desynchronization seen under placebo, especially in right frontal regions.

**Conclusion:**

A single 10 mg dose of MPH homogeneously modulates frontal α power during a visuomotor task, promoting sustained cortical activation. This paradigm emerges as a sensitive tool for studying motor-cognitive coupling and may contribute to understanding MPH mechanisms in both healthy and clinical populations.

## INTRODUCTION

Alpha power, a crucial component of electroencephalographic (EEG) activity, plays a significant role in facilitating motor–cognitive tasks by modulating attentional resources and sensorimotor integration. 1 Studies have shown that increased α band activity is associated with improved performance in tasks that require precise coordination between visual input and motor output, suggesting that α oscillations function as a means of neural regulation, optimizing the brain's readiness to process information effectively. 2

In the context of methylphenidate (MPH) ingestion, which is known to enhance dopaminergic activity and potentially alter cortical rhythms, understanding the acute effects on α power becomes vital. 3 For instance, alterations in α connectivity have been linked to the transient formation of functional networks that support visuomotor coordination during task execution, highlighting the relevance of α power in predicting performance outcomes. 4

Thus, examining these dynamics in healthy subjects offers valuable insights into the interplay between neurotransmitter modulation and cognitive task execution. As a commonly used psychostimulant, MPH aims to enhance cognitive abilities in various social contexts. One of its most cited properties is the inhibition of dopamine reuptake by blocking the dopaminergic transporter, allowing for its increased concentration in the synaptic cleft. 5

The use of MPH enhances alertness and attention by acting on the central nervous system (CNS), reducing both physical and mental fatigue while increasing arousal levels. 6 Different results demonstrated the modulatory effects of MPH on cortical and subcortical areas. These regions are related to attention, impulsiveness, and alertness. 7 8 9 Executive functions generally have a high affinity with the prefrontal cortex (PFC), striatum, and nucleus accumbens. 10 11 12

The results regarding the impact of MPH on cognitive functions are inconclusive. Some studies could not prove that the drug improves learning or planning. 13 14 15 Dyme et al. found no evidence supporting its effectiveness in improving cognitive functions related to learning. 16 Another experiment reported that the drug enhanced retrieval of information from memory, at longer intervals, specifically in situations where there were already impediments (e.g., the age-dependent decline in memory). 17 In this direction, several findings report that MPH does not drastically improve the formation of new memories in healthy individuals. It can still improve long-term retention if taken after the information has already been acquired. 18

## METHODS

A total of 13 healthy individuals participated in the study, 7 of whom were men and 6 women, with an average age of 25.6 (standard deviation [SD]: 4.5). The exclusion criteria were patients with mental or physical illness and a history of psychoactive or psychotropic substance use. For this reason, all participants went through anamnesis and a clinical exam. All individuals were right-handed, in agreement with the Edinburgh inventory. 19 Additionally, they were not allowed to sleep for less than 6 to 8 hours on the night before the experiment. All subjects signed an informed consent form and were aware of the experimental protocol approved by the ethics committee of the university, under the CAAE: 16342213.4.0000.5263.

### Data acquisition and processing

We recorded the electroencephalography signal acquisition using the 20-channel Braintech-3000 EEG system (EMSA Medical Instruments). A spandex cap with 20 electrodes (EletroCap Inc.) was used, according to the International 10/20 system for electrodes. Then, two more electrodes were positioned on the earlobes, yielding 20 mono-polar derivations (Fpz as a ground electrode). 20 Furthermore, we attached two 9 mm-diameter electrodes above and on the external corner of the right eye in a bipolar electrode montage to monitor artifacts on eye movements, that is, electrooculography (EOG).

Electrode impedance for EEG and EOG was limited to less than 5 kΩ. The data acquired had a total amplitude of less than 100 μV. The EEG signal was amplified, with a gain of 22,000, analogically filtered between 0.1 and 100 Hz, and sampled at 240 Hz. For the reference data analysis, we applied a visual exam and an independent component analysis (ICA) to eliminate the possible sources of artifacts produced by the task. 21

We excluded data from individual electrodes which lost contact with the scalp, or which showed high impedance (> 10 kΩ), as well as data from blocks with movement artifact excess (±100 µV). Then, we applied ICA to identify and remove any remaining artifacts after the initial visual inspection. As a group of blind source separation methods (spatial filters), ICA aims to estimate the maximum independent components from a statistical point of view. We removed independent components resembling blinking or muscle artifacts.

Using established procedures, we projected the remaining components onto the scalp electrodes by multiplying the input data by the inverse matrix of the spatial filter coefficients derived from ICA. We utilized the earlier rejection standards, and the ICA-filtered data were then reinspected for residual artifacts. Using MATLAB (MathWorks Inc.), we applied a traditional estimator for the power spectral density (PSD) or directly from the square modulus of the Fourier transform.

### Experimental procedure

We minimized sensory interference, and the subjects performed the task in a sound and light-attenuated room. Each subject underwent two experimental conditions: placebo (PL) and 10 mg of MPH, following a randomized, double-blind design on 2 days. Each subject executed one condition on the first day and the other on the second day, 1 week apart. The participants sat on a comfortable chair with armrests to minimize muscular artifacts while we recorded the EEG data before, during, and after the motor task. All individuals had to be familiar with the experimental task.

The procedures were standardized in the following sequence: application of neuropsychological tests and EEG recording at rest. The neuropsychological tests were administered prior to MPH or placebo ingestion. First, we collected EEG data for each subject for 3 minutes (eyes open). After the capsule ingestion, subjects rested for 2 hours (i.e., drug peak level). Under both conditions, the subject performed 6 blocks of 15 trials each (Aim Task, as explained in the Experimental Task section). Then, the subjects ingested a capsule containing a placebo or MPH and rested for 2 hours. After that, we recorded another 3 minute EEG signal acquisition with open eyes.

The exact sequence was then repeated (rest EEG/aim task/rest EEG) with participants on the drug. The inclusion of neuropsychological assessments aimed to evaluate baseline cognitive functions such as attention, working memory, and inhibitory control, which are relevant to the MIRA task's demands. These assessments provided a comprehensive profile of participants' cognitive abilities prior to the intervention, ensuring that any observed effects could be attributed to MPH rather than preexisting cognitive differences.

### Experimental task

The subject held a joystick with the right hand (Quick Shot-Crystal CS4281) in front of a monitor while the other remained at rest. He viewed from the screen, with a black background, a cross (crosshairs) from the monitor that appeared randomly on the screen in each attempt. After 2 seconds, the cross disappears, and a target (i.e., a small circle) appears that moves in a zigzag (from left to right or from right to left on the monitor). Furthermore, the moment it passes through where the cross appeared, the subject presses the joystick's fire button.

A total of six task blocks were performed, with ten stimuli in each block. This is a motor-cognitive task requiring cognitive resources such as concentrated attention, spatial working memory, inhibitory control, and temporal perception. There is a randomness and unpredictability in the appearance of the cross between the right and left sides, minimizing the learning effect.

### Electroencephalographic measure

We analyzed absolute α power through a classic estimator for the power spectral density (PSD) or directly from the square modulus of the Fourier Transform (F.T.). We used the full α band, which refers to EEG oscillations in the 8 to 12 Hz frequency range, often associated with relaxed wakefulness and inhibition of cortical activity. Alpha activity describes the presence and patterns of these oscillations, while α power quantifies the amplitude or intensity of α oscillations, typically measured as the square of the signal's magnitude in the frequency domain.

### Cortical area and EEG frequency

Our analysis of executive functions in the frontal region was due to their close association. Attention, focus, planning, and short-term memory processes were the subjects of our study. The α frequency range was chosen because of its association with sensory experience, mental activity, and imagery. 22 23 24 25 The α band has been used as a marker for a relaxed state and activity reduction (α-blocking) as sensory stimulation or mental activity. 26 Activity in the α band increased with task practice, which might be related to the consolidation of task-related skills. 27

### Statistical analysis

We represented the results as Mean and SD. We implemented a two-way analysis of variance (ANOVA) to observe the interaction between conditions (placebo or MPH 10 mg) and moments (pre- and post-joystick button press). The factor “Moment” refers to pre- versus post-joystick button. Additionally, we paired *t* -tests to compare the moments (pre versus post) within each condition, aiming to explore the interaction. The significance criterion was *p* -value ≤ 0.05 for all analyses. We analyzed each electrode separately to avoid Type I errors. ANOVA 2-way: Placebo or MPH, and Drug versus Moment (before and after the treatment). We used a two-sample Student's *t* -test ( *p* ≤ 0.05) to compare the placebo and MPH treatment conditions. The Bonferroni correction adjusts probability ( *p* ) values because of the increased risk of a Type I error when making multiple statistical tests.

## RESULTS

### EEG parameters

We analyzed the absolute power of α in the prefrontal and frontal regions in the electrodes Fp1, Fp2, F7, F3, Fz, F4, and F8. The statistical analysis aimed to examine putative interactions and the respective main effects of condition and moments. The results of electrophysiological parameters were expressed through quantitative EEG (qEEG), absolute α power, and a 2-factor ANOVA (Conditions vs. Moments) performed to detect differences.

The condition factors contain two sub-levels: placebo and MPH; as do moment factors: before and after pressing the joystick during placebo or MPH condition. Whenever an interaction was identified, we performed paired *t* -tests (Student) to examine it within each group in greater detail.

**Figure 1:**
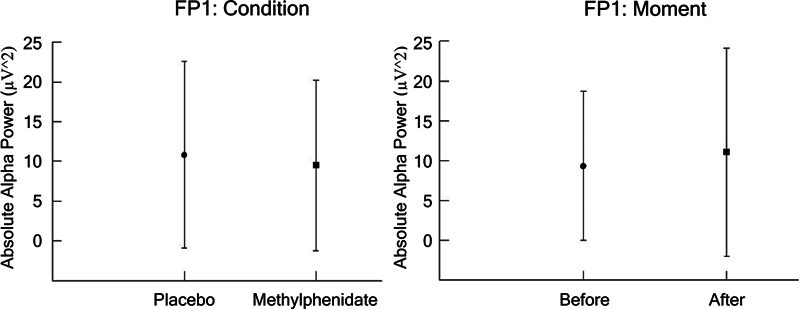
( **A** , **B** ) The FP1 electrode (mean ± standard deviation [SD]) for the dependent parameter absolute alpha power (µV ^2^ ), based on analysis of variance (ANOVA; condition versus moment). The results indicated a main effect for condition ( *p* = 0.011) and moment ( *p* = 0.001).

In the dependent parameter Fp1, the 2-way ANOVA (Group vs. Moment), we found a main effect for condition (F [1,1634] = 6.501; *p* = 0.011; η ^2^ *p* = 0.004; power = 72.2%) and moment (F [1,1634] = 11.122; *p* = 0.001; η ^2^ *p* = 0.007; power = 91.5%), as presented in Figure 1 .

**Figure 2:**
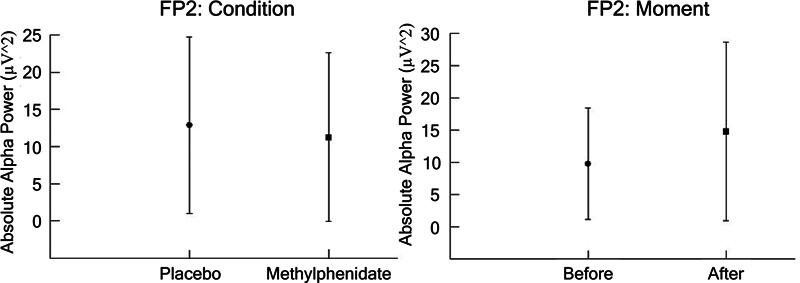
( **A** , **B** ) The FP2 electrode (mean ± SD) for the dependent parameter absolute alpha power (µV ^2^ ), based on ANOVA (condition versus moment). The results indicated a main effect for condition ( *p* = 0.000) and moment ( *p* = 0.002).

In Fp2, we found a main effect for condition (F [1,1640] = 10.273; *p* = 0.000; η ^2^ *p* = 0.047; power = 99.9%) and moment (F [1,1640] = 80.805; *p* = 0.002; η ^2^ *p* = 0.002; power = 86.5%), as shown in Figure 2 .

**Figure 3:**
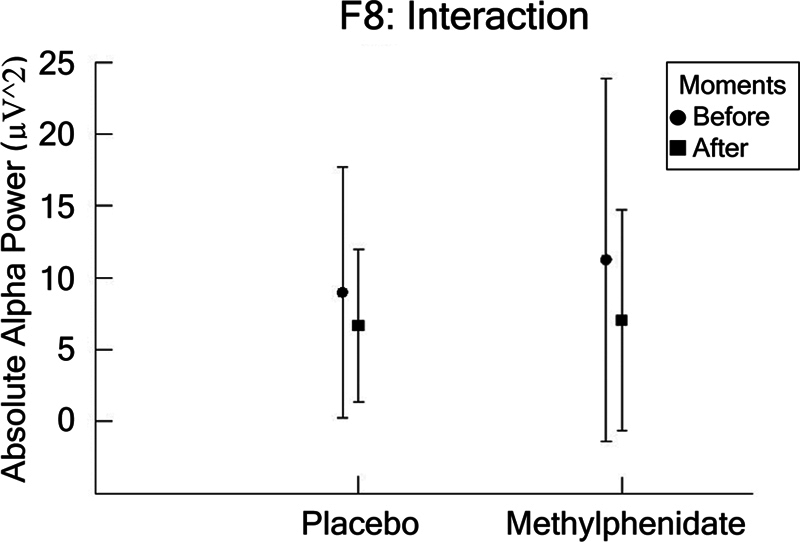
The F8 electrode (mean ± SD) for the dependent parameter absolute alpha power (µV ^2^ ), based on ANOVA (condition versus moment). The results indicated an interaction between condition and moment ( *p* = 0.023). A detailed inspection of the interaction was performed through a paired *t* -test within each condition between the pre- and postmoments for placebo ( *p* = 0.000) and methylphenidate (MPH; *p* = 0.254). Statistical significance is represented by an asterisk.

In F7, no significant differences were found. In the dependent variable F8, we found an interaction between condition and moment (F [1,1777] = 5.172; *p* = 0.023; η ^2^ *p* = 0.003; power = 62.3%). Observing the interaction in more detail, a paired *t* -test was performed to compare the behavior of the moments in each condition. The results showed differences at the time points for two conditions: placebo (t = −3.550; df = 415; *p* = 0.000) and MPH (t = −1.143; df = 434; *p* = 0.254), as shown in Figure 3 .

**Figure 4:**
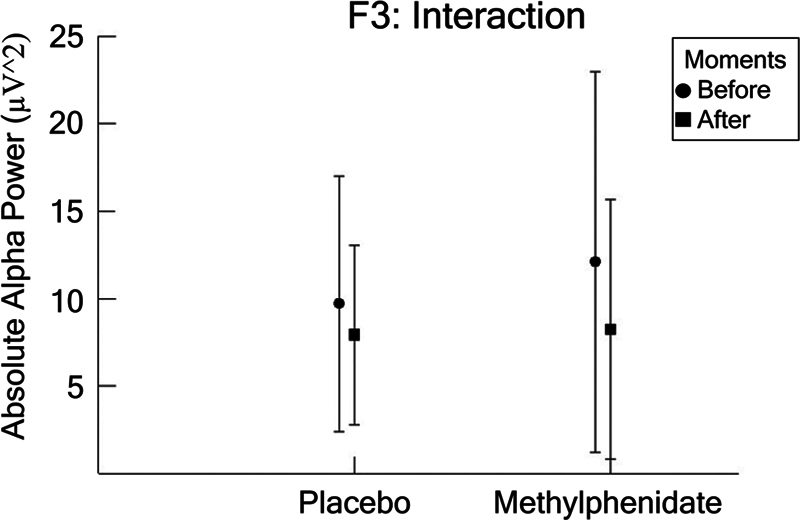
The F3 electrode (mean ± SD) for the dependent parameter absolute alpha power (µV ^2^ ), based on ANOVA (condition versus moment). The results indicated an interaction between condition and moment ( *p* = 0.006). A detailed inspection of the interaction was performed through a paired *t* -test within each condition between the pre- and post-moments for placebo ( *p* = 0.000) and MPH ( *p* = 0.191). Statistical significance is represented by an asterisk.

In F3, we found an interaction between condition and moment (F [1,1782] = 7.603; *p* = 0.006; η ^2^ *p* = 0.004; power = 78.7%). Observing the interaction in greater detail, we performed a paired *t* -test to compare the behavior of the moments in each condition. The results showed differences at the time points for two conditions: placebo (t = −3.741; df = 419; *p* = 0.000) and MPH (t = −1.309; df = 432; *p* = 0.191), as presented in Figure 4 .

**Figure 5:**
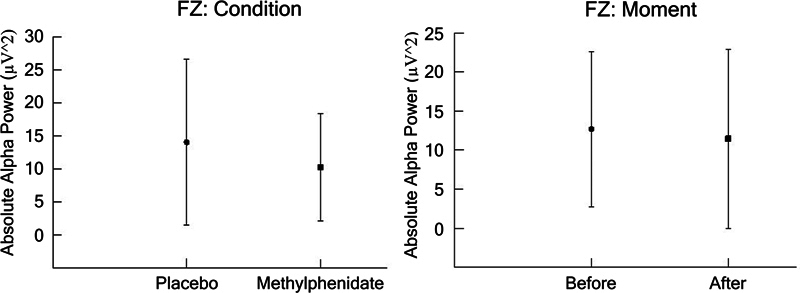
( **A** , **B** ) The FZ electrode (mean ± SD) for the dependent parameter absolute alpha power (µV ^2^ ), based on ANOVA (condition versus moment). The results indicated a main effect for Condition ( *p* = 0.000) and Moment ( *p* = 0.002).

In Fz, we found a main effect for condition (F [1,1733] = 55.644; *p* = 0.000; η ^2^ *p* = 0.031; power = 99.9%) and moment (F [1,1733] = 4.035; *p* = 0.045; η ^2^ *p* = 0.002; power = 51.9%), as shown in Figure 5 .

**Figure 6:**
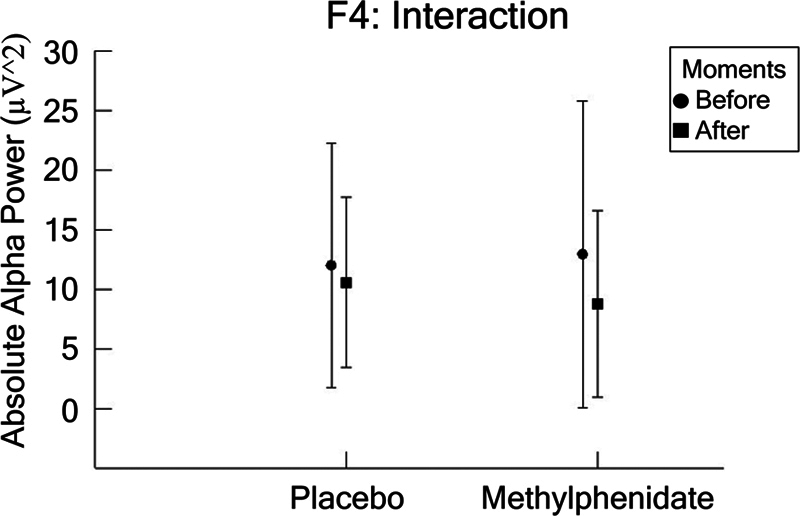
The F4 electrode (mean ± SD) for the dependent parameter absolute alpha power (µV ^2^ ), based on ANOVA (condition vs. moment). The results indicated an interaction between condition and moment ( *p* = 0.005). A detailed inspection of the interaction was performed through a paired *t* -test within each condition between the pre- and post-moments for placebo ( *p* = 0.000) and MPH ( *p* = 0.032). Statistical significance is represented by an asterisk.

In F4, we found an interaction between condition and moment (F [1,1752] = 8.638; *p* = 0.003; η ^2^ *p* = 0.005; power = 83.6%). Observing the interaction in greater detail, we performed a paired *t* -test to compare the behavior of the moments in each condition. The results showed differences at the time points for two conditions: placebo (t = −4.233; df = 412; *p* = 0.000) and MPH (t = 2.147; df = 432; *p* = 0.032), as shown in Figure 6 . Table 1 has a summary of our findings.

**Table 1:** Summary of statistical results for absolute α power in frontal electrodes under placebo and methylphenidate (MPH) conditions

Cortical region	Electrode	Hemisphere	Condition/time and interaction effects on alpha power	Interpretation regarding cortical activity
Prefrontal	Fp1	Left	Main effect for condition (F = 6.501, *p* = 0.011) and moment (F = 11.122, *p* = 0.001)	Reduced alpha power under MPH, indicating increased cortical activation
Prefrontal	Fp2	Right	Main effect for condition (F = 10.273, *p* = 0.000) and moment (F = 80.805, *p* = 0.002)	Reduced alpha power under MPH with greater variability, suggesting enhanced engagement but potential instability
Inferior prefrontal gyrus	F7	Left	No significant difference	No notable MPH effect
Anterior frontal	F3	Left	Interaction (F = 7.603, *p* = 0.006); placebo: t = −3.741, *p* = 0.000; MPH: t = −1.309, *p* = 0.191	MPH attenuates pre–post differences, suggesting sustained activation
Medial frontal	Fz	Medial	Main effect for condition (F = 55.644, *p* = 0.000) and moment (F = 4.035, *p* = 0.045)	General reduction in α power under MPH
Anterior frontal	F4	Right	Interaction (F = 8.638, *p* = 0.003); placebo: t = −4.233, *p* = 0.000; MPH: t = 2.147, *p* = 0.032	MPH leads to increased alpha postpress, indicating engagement of right anterior frontal cortex after response
Inferior prefrontal gyrus	F8	Right	Interaction (F = 5.172, *p* = 0.023); placebo: t = −3.550, *p* = 0.000; MPH: t = −1.143, *p* = 0.254	MPH modulates response, reducing pre–post differences

## DISCUSSION

Our experiment aimed to observe the effect of MPH in areas of the frontal cortex during the performance of a visuomotor targeting task. The results indicated a reduction in the absolute potency values in α in the MPH condition when compared with placebo, bilaterally in the prefrontal area (electrodes Fp1 and Fp2), unilaterally in the right inferior prefrontal gyrus (electrode F8), and bilaterally in the anterior frontal area (electrodes F3, Fz, and F4). Several results suggest a greater affinity of MPH with the PFC. 10 11 12

The PFC plays a central role in cognitive control functions, which is responsible for various executive functions. This region can orchestrate the parts dynamically to achieve a particular goal. Thus, the PFC recruits other subregions synchronized with the ongoing main target. The PFC is associated with cognitive and motor action planning, decision-making, problem-solving, general inhibitory control, speech and language regulation/control, judgment ability, working memory, cognitive flexibility, and even different personality types. 28

In this sense, the experimental task used in the present study required many of these functions, justifying their involvement. As for the inferior prefrontal gyrus, there is a robust relationship between this region and aspects related to language and semantics. This relationship was more evident in experiments with lesions. 29

The targeting experimental task imposes on the subjects an inhibitory control of the firing action until the target stimulus reaches the correct position, which must be kept in the spatial working memory. Our results suggest that MPH caused a change in the behavior of this region. The absence of a significant difference between MPH and placebo in the left region (electrode F7) may indicate a functional asymmetry between the left and right inferior prefrontal gyrus, in which the former does not have the same affinity for the drug's effect and performs less relevant functions during the experimental task employed in this research. 30

The anterior frontal area represents a considerable portion of the frontal cortex; its cytoarchitecture refers to the frontal portion of the lobe of Guenon. This region is also known as the frontal visual field (FVC). This denomination is due to its participation in the mechanisms of eye movement control. Our results suggest an generalized increased activity (electrodes F3, Fz, and F4) in the anterior frontal area. Several studies associate this area with a wide range of executive functions, such as learning and motor imagery, executive behavior control, planning, and working memory.

Notably, the right lateral region of the FVC is involved with aspects of spatial and visuomotor attention. Still, it exerts regulation in the control of top-down attention. 31 Data extracted from monkeys have shown that the FVC has conglomerates of neurons that fire in response to changes in covert spatial attention, and those microstimulations of the FVC show an improvement in attention in areas contralateral to stimulation. 32 Experiments using transcranial magnetic stimulation (TMS) in the right region of the FVC produce a significant distracting increase on both sides of the spatial field (right and left).

Given the characteristics of the aiming task, that is, the need for visual monitoring of target stimuli moving randomly from both the left and right sides of the monitor, we can suggest that MPH could influence spatial attention. The FP2 showed greater variability in α power responses under MPH, potentially reflecting individual differences in dopaminergic modulation or task engagement. In F4, both conditions showed pre- to post-task differences, with MPH producing a marked α decrease following joystick pressing, indicating an engagement of the right anterior frontal cortex after motor response under MPH influence.

A limitation of the present study is the potential practice effect from the first to the second session. However, MPH does not appear to differentially influence this effect compared with placebo, as the task's randomness minimizes learning.

In conclusion, the present study confirmed the crucial modulatory effect of MPH in the prefrontal areas, as suggested by other studies. The use of qEEG proved to be invaluable for capturing electrophysiological changes associated with the action of psychostimulant substances such as MPH.

The α frequency band was significantly sensitive to the effect of MPH, helping to differentiate its action from cortical areas involved with relevant cognitive processes: concentrated spatial attention, visuomotor integration, spatial working memory, inhibitory control of action, and control of eye movements. Thus, such processes continue to be investigated from other specific experimental tasks to complement the findings obtained through the aiming task.

The paradigm used here would also be helpful for studies with subjects with attention deficit hyperactivity disorder. This would include behavioral measures in addition to electrophysiological variables, since such measures from the aiming task can help to elucidate aspects of the symptoms of agitation, lack of concentration, and impulsiveness.

In this study, we examined the relationship between a sensorimotor integration task (“MIRA”) when participants were under the influence of MPH. We centered on the modulatory effects of the drug in a task involving elementary cognitive aspects, such as working memory, inhibitory control, and attention. The findings demonstrated that this drug affects cortical areas homogeneously, especially in the prefrontal region. Our results suggest experimental “pathways” for future studies. Researchers can use MPH to examine the coupling between motor versus cognitive processes, which were previously underexamined. Considering that researchers designated motor knowledge as implicit memory in the early 1950s, it appears that MPH can provide a “window” of opportunity to explore this relationship.
